# Cell behaviors within a confined adhesive area fabricated using novel micropatterning methods

**DOI:** 10.1371/journal.pone.0262632

**Published:** 2022-01-14

**Authors:** Tsukasa Nakatoh, Takuji Osaki, Sohma Tanimoto, Md. Golam Sarowar Jahan, Tomohisa Kawakami, Kentaro Chihara, Nobuyuki Sakai, Shigehiko Yumura

**Affiliations:** 1 Graduate School of Sciences and Technology for Innovation, Yamaguchi University, Yamaguchi, Japan; 2 Shoyo Sangyo Co., Ltd, Chuo-ku, Osaka, Japan; 3 Department of Biochemistry and Molecular Biology, University of Rajshahi, Rajshahi, Bangladesh; University of Sharjah, UNITED ARAB EMIRATES

## Abstract

In the field of cell and tissue engineering, there is an increasing demand for techniques to spatially control the adhesion of cells to substrates of desired sizes and shapes. Here, we describe two novel methods for fabricating a substrate for adhesion of cells to a defined area. In the first method, the surface of the coverslip or plastic dish was coated with Lipidure, a non-adhesive coating material, and air plasma was applied through a mask with holes, to confer adhesiveness to the surface. In the second method, after the surface of the coverslip was coated with gold by sputtering and then with Lipidure; the Lipidure coat was locally removed using a novel scanning laser ablation method. These methods efficiently confined cells within the adhesive area and enabled us to follow individual cells for a longer duration, compared to the currently available commercial substrates. By following single cells within the confined area, we were able to observe several new aspects of cell behavior in terms of cell division, cell–cell collisions, and cell collision with the boundary between adhesive and non-adhesive areas.

## Introduction

In the field of cell and tissue engineering, there is an increasing demand for techniques to place and arrange cells to adhere to substrates of desired shapes and sizes. In applied medicine, in particular, cell sheets of specific shapes and sizes are essential for repairing the skin and cornea, and this material is also critical for transplants in regenerative medicine. In addition, in basic research, local control of cell adhesion has proven to be an efficient tool for studying cell migration, dynamics of cell adhesion, cell spreading, and cell–cell interactions [1–4]. Confinement of single cells for adhesion to small, geometric patterns can also be a powerful tool to artificially control cell shape and cytoskeletal organization [5–7]. Although recent advances in materials science have included topographical fabrication with 3D architecture to adhere cells [8–11], there is still demand for 2D fabrication.

The earliest methods for adhering cells to a confined area of substrate were designed in the 1960s. Coverslips were coated with a cellulose acetate film, and a palladium coating was then added by evaporation through a hole of the desired shape in a nickel mask sheet. In this approach, there is limited attachment of the cells to the area of the palladium coating [12, 13]. More recent methods for construction of cellular patterns include soft lithography [14, 15], inkjet printing [16], dip-pen nanolithography [17, 18], photolithography [19], and microserigraphy [20]. An overview and details of the fabrication methods are available in a number of excellent reviews [6, 10, 11, 21–24]. All of these methods involve printing—at the nanometer to micrometer scale on coverslips—of positively charged proteins (such as polylysine), extracellular matrix proteins (such as collagen, fibronectin, and laminin), or synthetic substances. These proteins and substances facilitate the adherence of cells to the patterned area.

These conventional methods have several drawbacks: they require special skills and expensive instruments (such as a special inkjet printer and an atomic force microscope), and the protocols are often time-consuming complicated, all of which limits the application of such methods in routine laboratory processes (such as the fabrication of stamps and photosensitive materials). The principle for most of these methods involves “positive” printing of cell-adhesion substances to promote the adhesion of cells to the printed area. However, most cultured animal cells, as well as cells of lower organisms such as *Dictyostelium*, can attach to the untreated surface of a coverslip or a plastic dish without any deficiency in growth or differentiation.

In the present study, we describe two novel methods for fabricating patterned adhesive substrates to confine cells. Both methods involve the simple “negative” principle. In the first method, the surface of the coverslip or plastic dish is coated with Lipidure, a non-adhesive coating material, and adhesiveness is localized on the surface by applying air plasma through a mask with holes. In the second method, after the surface of the coverslip is coated with gold by sputtering and then with Lipidure, the Lipidure coat is locally removed with a scanning laser ablation by applying a recently developed laserporation method [25–27]. Both of these methods are very simple, time-saving, inexpensive, and do not require special skills; thus, technicians can efficiently confine cells within the adhesive area for a longer time than with commercially available substrates. To date, micropatterns have not been used to study cell division, cell–cell collision, and cell collision with the boundary between the adhesive and non-adhesive substrate. By using these novel methods, we observed several notable aspects of cell behavior.

## Materials and methods

### Cell preparation

*Dictyostelium* cells (AX2) were cultured in plastic dishes at 22°C in HL5 medium (1.3% bacteriological peptone, 0.75% yeast extract, 85.5 mM D-glucose, 3.5 mM Na_2_HPO_4_, and 3.5 mM KH_2_PO_4_, pH 6.3), as previously described [28]. The Simian-virus-40-transformed African green monkey kidney cell line, Cos-1, was maintained in plastic dishes containing Dulbecco’s modified Eagle’s medium (Gibco BRL, Thermo Fisher Scientific, Japan) supplemented with 10% fetal bovine serum (Gibco BRL).

### Plasmids and transformation

The GFP (green fluorescent protein)-lifeact expression vector has been previously described [29]. The vector was transformed into cells through electroporation or laserporation, as described previously [27, 30]. The transformed *Dictyostelium* cells were selected in HL5 medium containing 10 μg·mL^-1^ G418 (Wako Pure Chemical Corporation, Osaka, Japan) in plastic dishes.

### Fabrication of micropatterned substrates using plasma and masks (Method 1)

The surface of the coverslip of a glass-bottom chamber or the bottom surface of a plastic (polystyrene) dish was coated with Lipidure, a water-soluble polymer of 2-methacryloyloxy ethyl phosphorylcholine (CM5206, NOF, Japan) [31]. First, the surface of the coverslip was treated with air plasma for 30 s using a plasma ion bombarder (PIB-10, Vacuum Device Inc., Japan). Ten microliters of 0.5% (w/v) Lipidure (dissolved in ethanol) were spin-coated onto the surface of a coverslip and then air-dried at 22°C. To coat the surface of the plastic dish (5.5 cm in diameter), it was treated with plasma for 30 s, and 1 mL 0.5% (w/v) Lipidure was then poured onto the surface. After incubation for 1 min, the excess solution was removed, and the samples were air-dried. Several types of masks were used: a nylon mesh (15 × 15 μm square mesh), aluminum foil with circular holes (100 μm in diameter, 12.5 μm thick), polyimide film with playing card suit-shaped holes (heart, diamond, club, and spade, 600 μm, 5 μm thick). The aluminum foil and polyimide film masks were custom-made by Shoyo Sangyo Co. Ltd. (https://www.shoyo-sangyo.co.jp). The mask was placed in contact with the surface of the Lipidure-coated coverslip, and air plasma was then applied from above in a chamber for 30 s. To visualize the Lipidure-coat, 5 μg·mL^-1^ of CellMask Orange (Thermo Fisher Scientific, Japan) in distilled water was incubated on the surface of the substrate for 5 min, following which the surface was washed three times with distilled water. A stock solution (1 mg·mL^-1^) of CellMask Orange was prepared in dimethyl sulfoxide. Cytographs (L10S300, Dai Nippon Printing, Tokyo, Japan) were used for comparison.

### Fabrication of micropatterned substrates by laser ablation (Method 2)

Coverslips were coated with gold (20 nm thick) using an ion-sputtering device (SC-708, Sanyu Electron Co., Ltd., Japan) and then coated with Lipidure. A laser beam was focused on the gold coat surface through a 20× objective lens under an inverted microscope (IX71, Olympus, Tokyo, Japan). A CW laser (100 mW-green DPSS laser, Active Ray Scientific Co. Ltd., Tokyo, Japan) was used. The focused beam removed the Lipidure coat, resulting in the appearance of a bright spot 0.5 μm in diameter. To generate the adhesive areas, the laser beam scan was controlled via a motorized x-y stage, operated using a computer. The graphic G-codes were generated using Inkscape software (http://inkscape.org), and the x-y stage was controlled using Universal G-code Sender software (https://github.com/winder/Universal-G-Code-Sender).

### Microscopy

*Dictyostelium* or Cos-1 cells were placed on the surface of a micropatterned substrate. After the suspension of cells were applied to the surface, the unattached cells were removed by changing the medium several times. Cells were observed under phase-contrast microscopy (IX-71, Olympus), and images were captured with a CMOS camera (DMK23UX249, Argo Corp., Osaka, Japan) at 10-s intervals. The images were processed using the ImageJ software (http://rsb.info.nih.gov/ij). The trajectories of cell migration and cell velocities were examined using the plugin Manual Tracking for ImageJ software. The fluorescence of CellMask Orange on the patterned surface was excited with a He/Ne laser (543 nm), and the emission was filtered with a long-pass filter (>560 nm) using a confocal microscope (LSM 510 Meta, Zeiss, Germany). This microscope was also used for simultaneous observations with differentiation interference, fluorescence microscopy, and interference reflection microscopy. Interference reflection microscopy was conducted as previously described [32, 33]. Fluorescence intensities were analyzed using ImageJ software.

To examine the doubling times of cell growth in shaking culture, cells were cultured in conical flasks (100 mL) containing 20 mL HL5 medium, with the reciprocal shaker operating at 150 rpm [31]. Cell density was measured using a hemocytometer. The cell growth in the adhesion areas on the fabricated coverslip was manually counted after capturing images for 12 h in the same microscopic field.

### Simulation using virtual rigid particles

The number of collisions in the circle versus the density of virtual rigid particles was calculated using the following equation:

$$Z=N\left(N-1\right)LV/2(A-NS)$$

where *Z* is the number of collisions, *N* is the number of particles, *L* is the diameter of the particle, *V* is the velocity of the particle, *A* is the area of the circle, and *S* is the area of the particle. *L*, *V*, *A*, and *S* were set at 8.42 μm, 234.6 μm·h^-1^, 7853 μm^2^, and 55.7 μm^2^, respectively.

### Statistical analysis

Statistical analyses were performed using GraphPad Prism 8 (GraphPad Software, Inc., San Diego, CA, USA). Data are presented as the mean ± standard deviation (SD) and were analyzed with Student’s *t*-test (for comparison between two groups) or with one-way ANOVA with Tukey’s multiple-comparison test.

## Results

### Novel methods to fabricate patterned adhesive substrates

**Fig 1:**
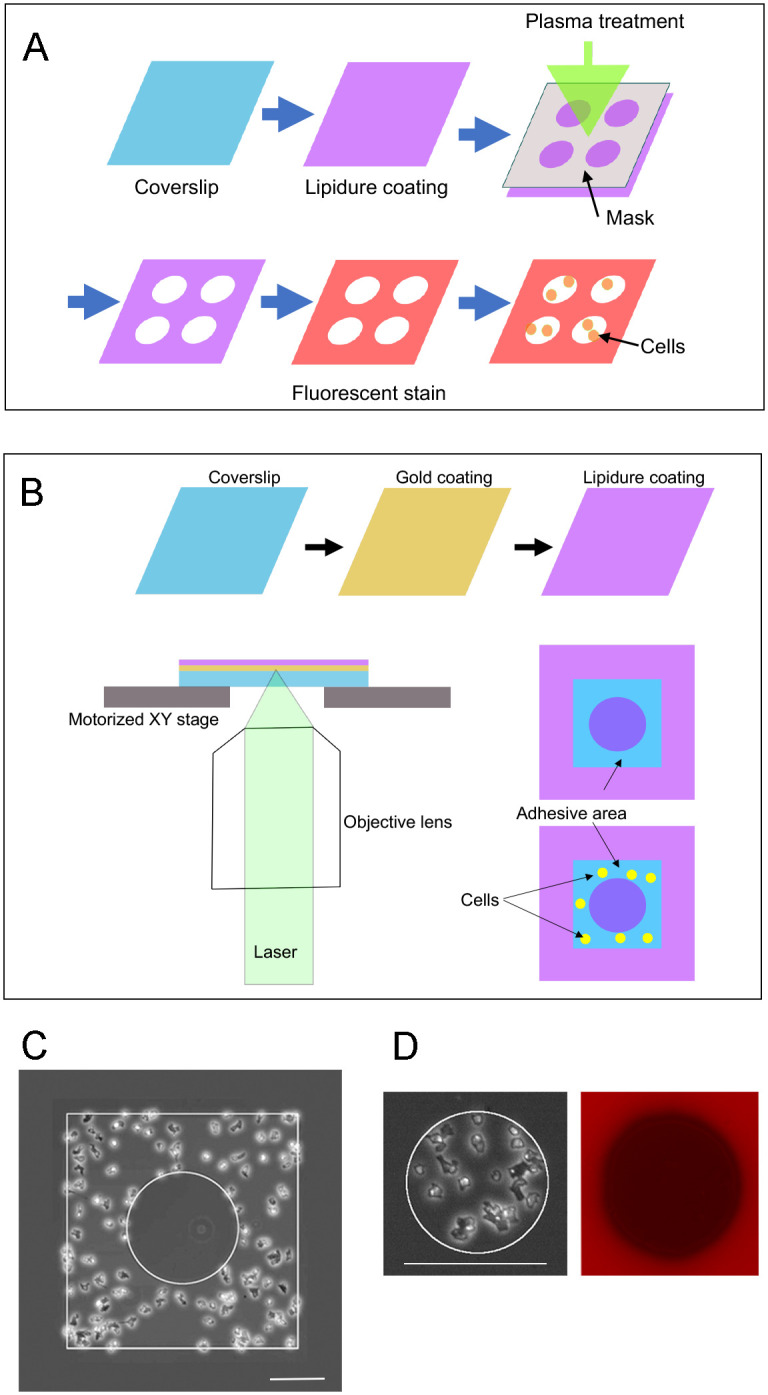
Novel methods to fabricate patterned adhesive substrates. (A) Overview of Method 1. Coverslips were coated with Lipidure. Air plasma was applied on the Lipidure-coated surface using a mask with holes in the target shapes (4 circular holes in this case). Only the plasma-applied areas became adhesive for cells. If necessary, the surface was stained with CellMask Orange, which visualized the adhesive area. Cells were then placed on the surface. Cells adhered only to the adhesive area. (B) Overview of Method 2. Coverslips were coated with gold by sputtering and then coated with Lipidure. A laser beam was focused on the gold coating through a 20× objective lens under a microscope. The laser beam scan was controlled using a computer-guided, motorized x-y stage. The Lipidure coat was removed by the laser and the laser-illuminated area became cell adhesive (blue area). (C) A typical phase-contrast image of the fabricated substrate, including an adhesive area between a square and a circle inside the square. (D) A typical phase-contrast and fluorescence image of the fabricated substrate after staining with CellMask Orange. Note that CellMask Orange stained only the masked area (non-adhesive area), and the black area became cell-adhesive.

In this study we describe two novel methods to confine and pattern cell adhesion on a 2-dimensional surface. The first method is illustrated in Fig 1A. The surface of the coverslip or plastic dish was coated with Lipidure, a non-adhesive coating material, which has the same structure as the phosphatidylcholine polar bases that form the cell membrane [34]. We previously reported that *Dictyostelium* cells did not adhere to the Lipidure-coated substrate [31]. Air plasma was applied to the Lipidure-coated surface through a mask with the desired pattern holes (four circles in Fig 1A) to decompose or denature the coat. Only the plasma-applied area became adhesive for the cells, and the entire process was completed within several minutes.

In Fig 1B we present the second method. The surface of the coverslip was coated with gold by sputtering and then coated with Lipidure. The laser beam was focused on the gold coated surface through an objective lens under a microscope. The energy of the beam was absorbed in the gold, and the Lipidure coat was removed only in the illuminated region. The scan of the laser beam was controlled by using a computer-guided motorized x-y stage. Without the gold coating, the laser did not remove the Lipidure coat. This second method enabled the generation of adhesive areas of any size and shape. For example, it was possible to generate an adhesive area between a square and a circle inside the square (Fig 1B and 1C). This is not possible using the first method.

The adhesive areas fabricated using the second method were detectable under brightfield microscopy. The adhesive areas fabricated with the first method could be visualized as black regions under fluorescence microscopy after staining with CellMask Orange, a lipophilic fluorescent dye (Fig 1D).

### Patterning of cells on the substrate

**Fig 2:**
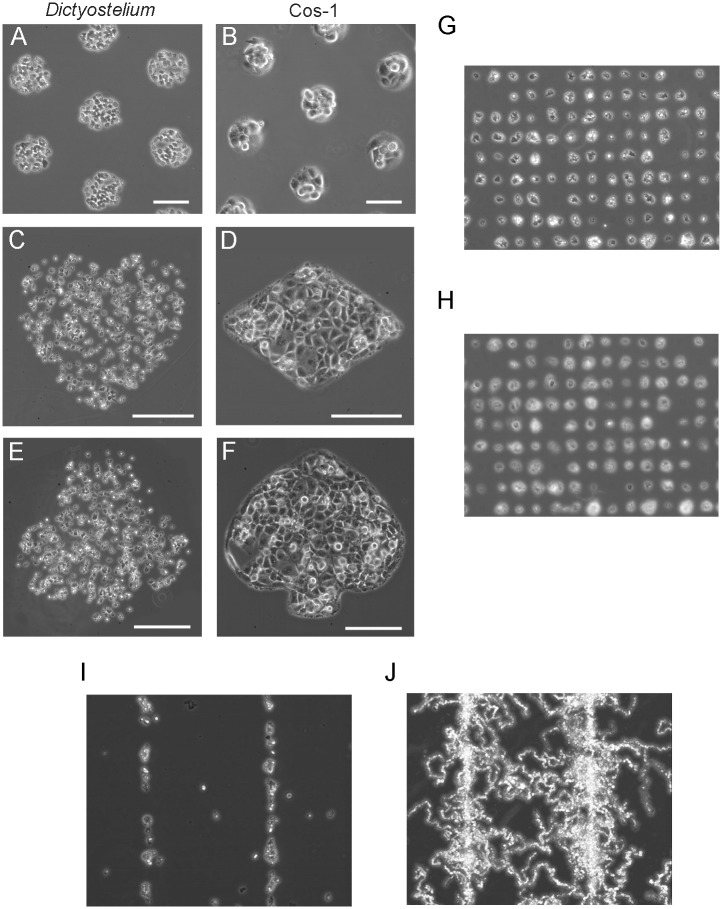
Patterning of cells on the substrate. (A) After *Dictyostelium* cells were placed on the substrate, they specifically adhered to the patterned adhesive areas (circles, 100 μm in diameter). Approximately 40–45 cells were observed within every single circle. (B) After Cos-1 cells were placed on the substrate, they also specifically adhered to the patterned adhesive areas (circles, 100 μm in diameter). Approximately 8–10 cells were observed within each circle. (C, D) *Dictyostelium* cells adhered within heart-shaped (C) and club-shaped (E) areas, and Cos-1 cells adhered within diamond-shaped (D) and spade-shaped (F) areas. Bars, 100 μm. (G) When a patterned surface was made by using a mask with a nylon mesh (15 x 15 μm square holes, 50 μm between holes), each single *Dictyostelium* cell was attached to each area and arranged in a latticework. (H) An averaged image (for 2 h) of the same view in panel G. (I) *Dictyostelium* cells were placed on a Cytograph with line-shaped adhesive areas (10 μm wide). The cells initially adhered to the adhesive lines to a greater degree. (J) An averaged image (for 2 h) of the same view in panel I. Note that the cells migrated away from the lines.

A suspension of *Dictyostelium* cells was placed on the surface with circular adhesive areas 100 μm in diameter. Cells attached only to adhesive areas, and the unattached cells showed Brownian motion (S1 Movie). After the unattached cells were removed by exchanging the medium, the remaining cells were found to be attached as islands (Fig 2A). Monkey kidney Cos-1 cells also generated similar islands (Fig 1B). When placed on the surface with adhesion areas having four playing card suit shapes (heart, diamond, club, and spade), the cells attached to these shapes (Fig 2C and 2E for *Dictyostelium* cells, and Fig 2D and 2F for Cos-1 cells).

When small adhesion areas (15 × 15 μm) were arranged in a lattice pattern, single cells were observed to be arranged into a lattice (Fig 2G). The averaged image from a 2-h observation period (Fig 2H) of the same view as in Fig 2G, shows that even with a longer observation period, individual cells were confined within the adhesive area.

Next, for comparing the new methods with previous ones, we used commercially available micropatterned adhesive coverslips manufactured using photolithography (Cytograph, L10S300, SD15, Dai Nippon Printing Co. Ltd., Tokyo, Japan). In Cytograph, the photoreactive polyethylene glycol (PEG)-coated coverslips were exposed to UV light through a photomask to activate the coated PEG and make it adhesive. *Dictyostelium* cells initially attached preferentially to the adhesive lines (Fig 2I) but gradually migrated away from the lines (Fig 2J). We conclude that using our proposed methods, cells were confined within the adhesive area for a longer time than with the commercially available substrates.

### Cell migration and cell division within a confined area

**Fig 3:**
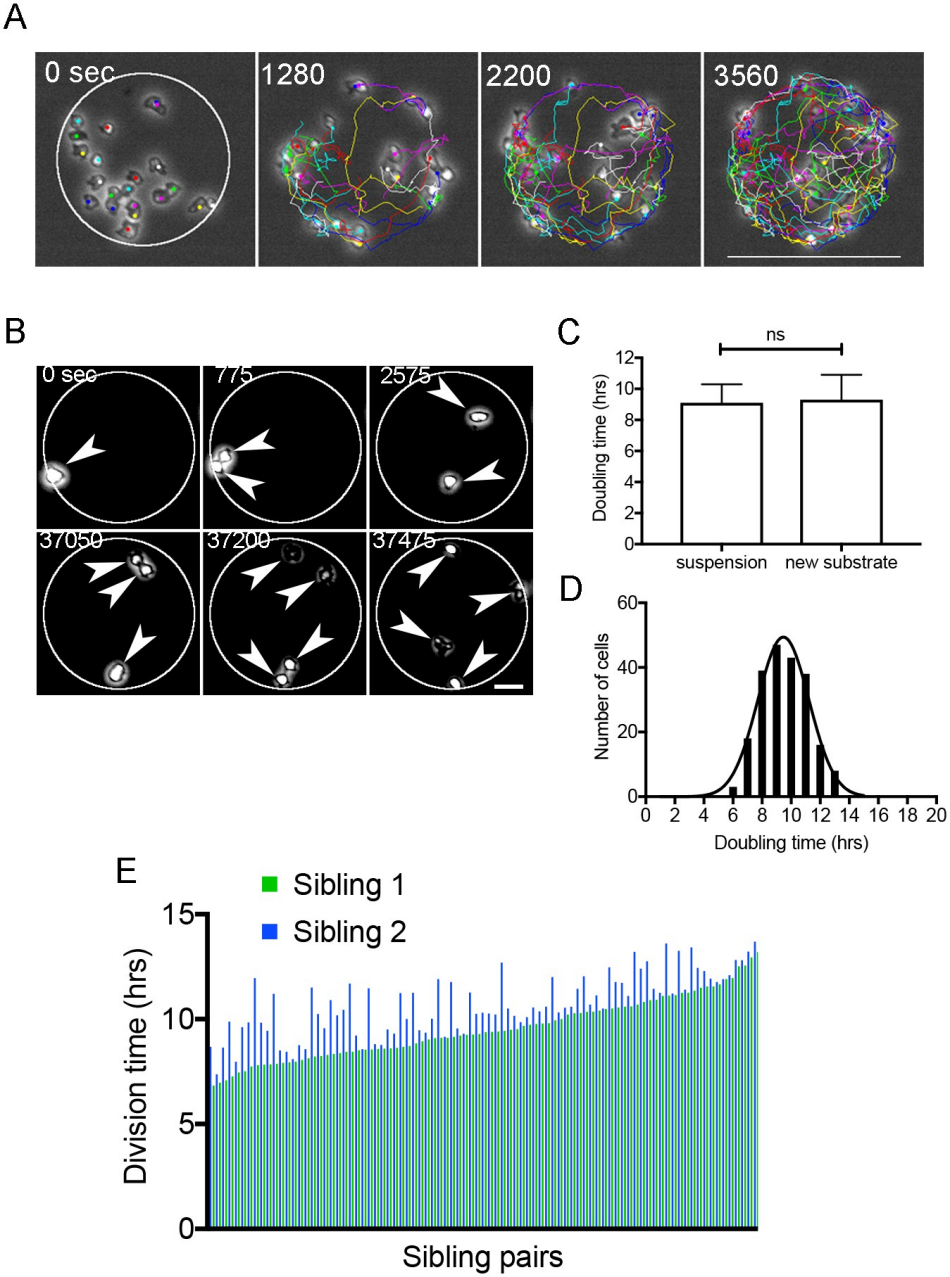
Cell migration and cell division within a confined area. (A) Typical cell trajectories for 1 h within the circle area. Bar, 100 μm. (B) Typical sequence of phase-contrast images of a single cell when it divided twice, i.e., into four granddaughter cells. (C) Comparison of doubling times, as measured both in a single-cell and by cell density (suspension culture). (D) Frequency of doubling times in the single-cell analysis. The curve shows the result of Gaussian fitting. (E) Division time for individual sibling pairs (individual pairs are shown in green and blue, n = 48 pairs).

To quantitatively characterize cell migration and cell division, it is necessary to follow individual cells after acquiring a timelapse movie. However, cells often migrate away from the microscopic field, which becomes a major challenge, especially when examining fast-migrating cells, such as *Dictyostelium*. In Fig 3A we show typical cell trajectories for 1 h within the circular area (100 μm in diameter).

It was also possible to follow events of cell division for a much longer time. In Fig 3B we show a typical sequence of phase-contrast images of a single cell when it divides twice, i.e., into four granddaughter cells.

The doubling time of cell growth has been conventionally examined by measuring the cell density of multiple cell populations (in suspension culture) using a hemocytometer. However, the present method enabled us to examine cell division at the single-cell level. Fig 3C presents a comparison between the doubling times with both single-cell measurement (9.3 ± 1.6 h, n = 212) and measurement via cell density (9.1 ± 1.2 h, 3 experiments by suspension culture); the results indicate that the doubling times were not significantly different between the two analyses. The frequency of doubling times in the single-cell analysis is shown in Fig 3D; the results indicate that the doubling times varied considerably among individual cells, with a typical Gaussian distribution (6–13 h). This type of observation cannot be achieved using conventional measurement methods.

Fig 3E presents the division times (from cell rounding to the abscission) for individual sibling pairs. Individual pairs are shown in green and blue (n = 48 pairs). The average difference was 1.03 ± 1.01 h, and the maximum difference was 4.15 h, suggesting that the siblings do not always synchronously divide. These observations have been challenging to achieve through conventional methods, especially for fast-moving cells.

### Collisions of migrating cells within a confined area

**Fig 4:**
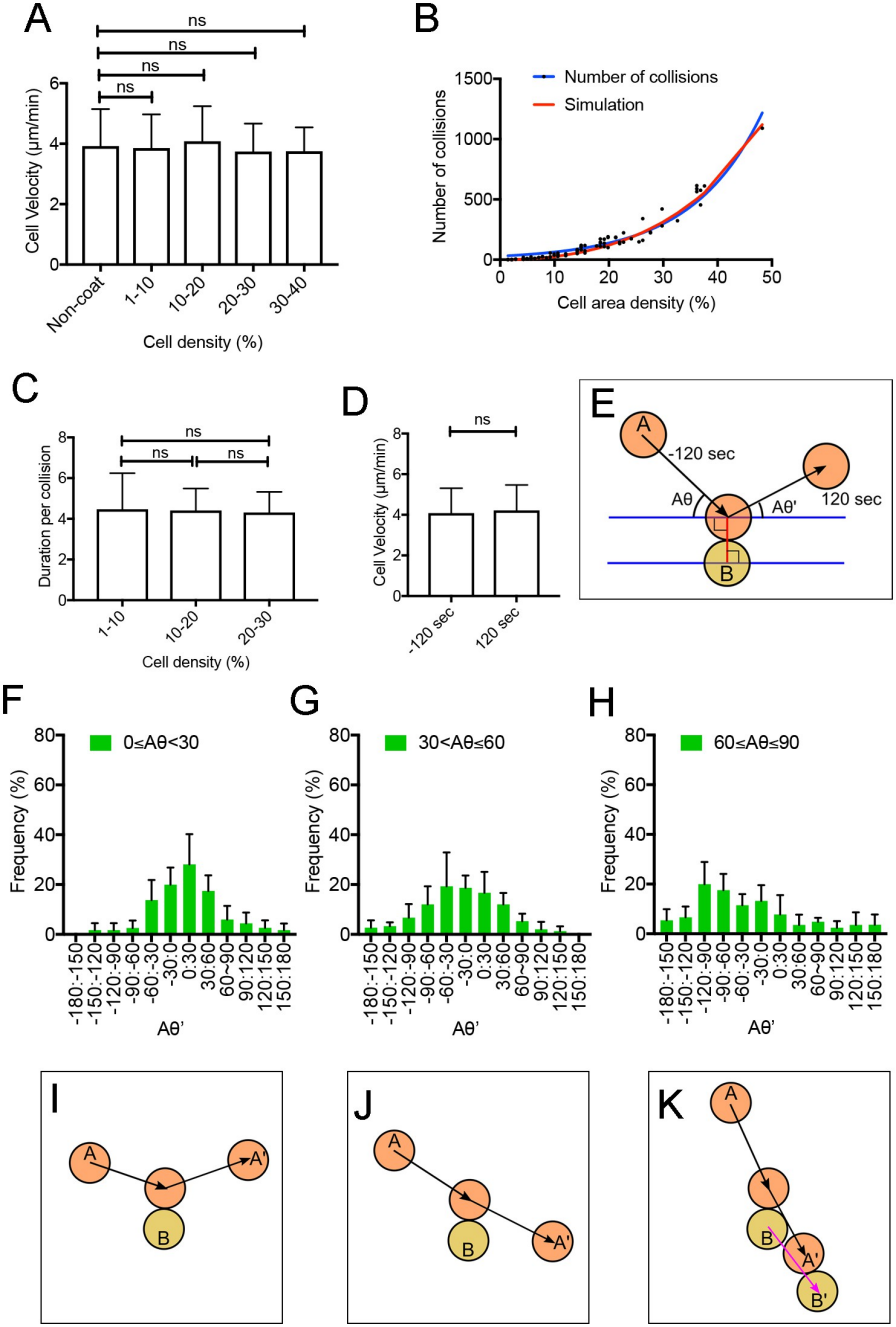
Collisions of migrating cells within a confined area. (A) Relationship between average cell velocity and cell density, defined as percentage of the total area of the circle occupied by cells. Velocity of freely migrating cells on non-coated coverslips was also plotted. Data are presented as mean ± SD. **P < 0.001; ns, not significant; P > 0.05. (B) Correlation between the number of cell collisions and cell density during 1 h of observation. The result of simulation using virtually moving rigid particles within the circle is shown in red. (C) Duration per cell collision versus cell density. Data are presented as mean ± SD. **P < 0.001; ns, not significant; P > 0.05. (D) Average velocity for 120 s before and after the collision. Data are presented as mean ± SD. **P < 0.001; ns, not significant; P > 0.05. (E) Explanation of angle measurement. When a migrating cell (A) collided with another cell (B), the entering angle (Aθ) and exiting angle (Aθ’) were measured. (F-H) Frequency of exiting angles when the entering angles were 0≤Aθ<30, 30<Aθ ≤60, and 60<Aθ≤90, respectively. (I-K) Depiction of migration direction when a cell (A) collides with another cell (B).

The average cell velocity within the confined area (3.9 ± 0.6 μm·min^-1^, n = 50) was not significantly different from that of freely migrating cells on untreated coverslips (4.0 ± 0.7 μm·min^-1^, n = 50) (Fig 4A). When multiple cells are placed within a confined area, the cells collide with each other, which may reduce their velocity. When cell density was increased to 40%, which was equivalent to approximately 250 cells in the circle (100 μm in diameter), the average cell velocity did not change (Fig 4A), suggesting that cell collision does not reduce the cell velocity. The cell density was defined as the percentage of the total area occupied by cells versus the circle area. Fig 4B presents the correlation between the number of cell collisions and the cell density during 1 h of observation: the number of cell collisions increased exponentially, in addition to an increase in the cell area density. We simulated this correlation using virtually moving rigid particles within the circle and found that cells collide with one another with the same frequency as do particles (red line in Fig 4B, see the details of the simulation in the Methods section). The duration of collision per cell does not change as the cell density increases (Fig 4C). The average velocity for 120 s before and after the collision is shown in Fig 4D; the results indicate that cells did not change velocity along with the cell–cell collisions.

Next, we examined the behavior of individual cells when cells collided with other cells. As depicted in Fig 4E, when a migrating cell (A) collided with another cell (B), the entering angle (Aθ) and exiting angle (Aθ’) can be measured. When entering at an angle lower than 30° (Aθ≤30), the cell tended to reflect against another cell, with a peak at 0≤Aθ’<30 (Fig 4F and 4I). When entering at an intermediate angle (30<Aθ≤60), the cell tended to pass through another cell (-60≤Aθ’<-30). (Fig 2G and 2J). When entering at an angle higher than 60° (60≤Aθ≤90), the cell tended to push or pass through another cell (-120≤Aθ’<-90) (Fig 4H and 4K).

### Cell behaviors at the boundary of the adhesive area

**Fig 5:**
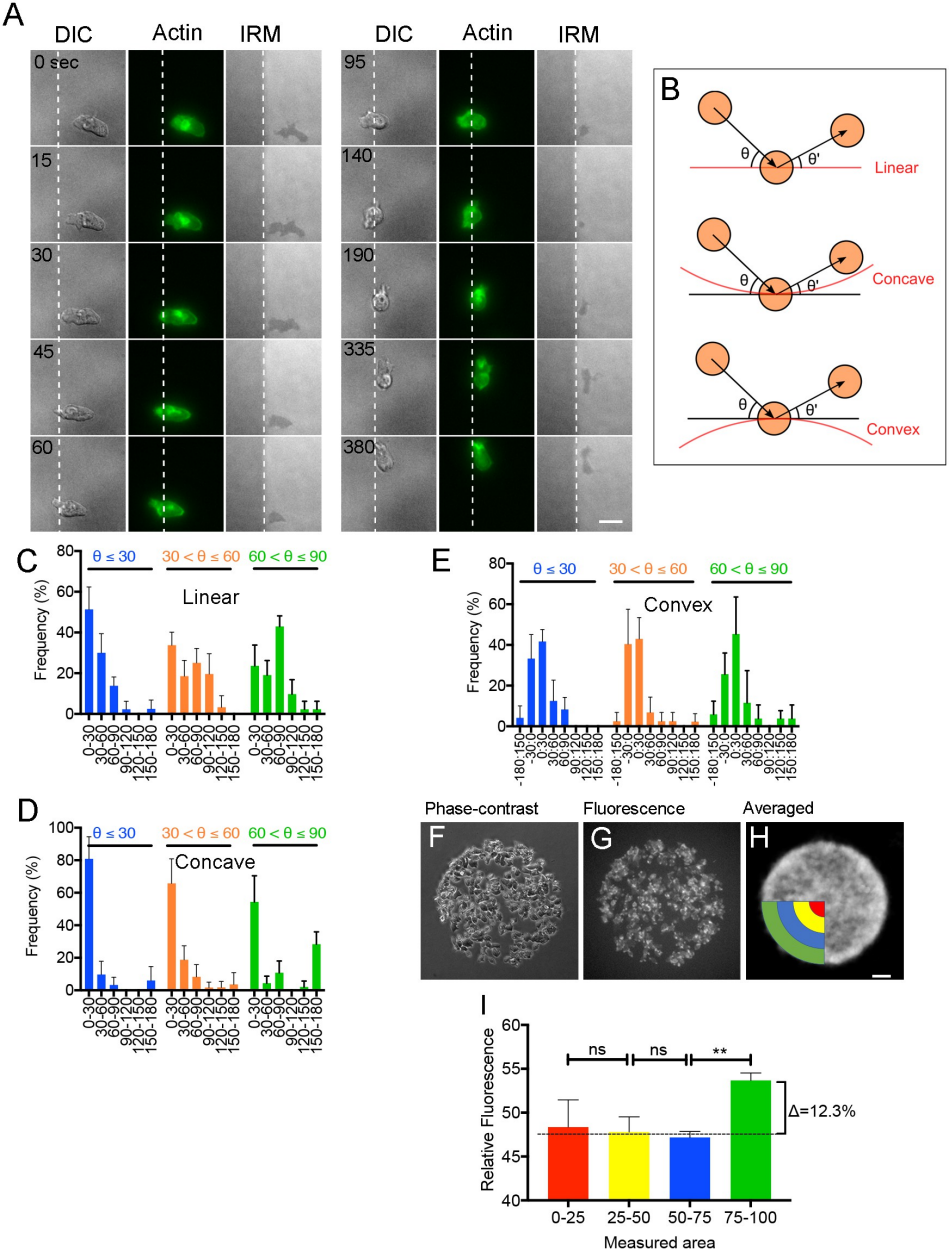
Cell behaviors at the boundary of the adhesive area. (A) Typical time series of images (DIC, fluorescence, and interference reflection microscopies) of a cell expressing GFP-lifeact. Dashed lines indicate the boundary between adhesive (right) and non-adhesive areas. (B) Depiction of the entering (θ) and exiting (θ’) angles when the cell encounters the following boundary (red) types: linear, concave arc, and convex arc. (C-E) Frequencies of exiting angles with entering angles of Aθ≤30, 30<Aθ≤60, and 60<Aθ≤90, when the cell encounters the linear, concave arc, and convex arc boundary, respectively. (F and G) Typical phase-contrast and fluorescence images of cells expressing GFP-lifeact within the circle (cell number, 200; area density, 32%), respectively. (H) The averaged fluorescence images for 1 h of panel G. Bar, 10 μm. (I) Average fluorescence intensity of the areas that were divided into 4 concentric regions (red, yellow, blue, and green in panel H). Data are presented as mean ± SD from 5 experiments. **P < 0.001; ns, not significant; P > 0.05.

Next, we examined the behavior of cells when they reached the boundary of the adhesive area. Fig 5A presents a typical time series of images (DIC, fluorescence, and interference reflection microscopy) of a cell expressing GFP-lifeact, a maker of F-actin. The dark region under interference reflection microscopy represents adhesion areas to the substrate. When a cell reaches the boundary (dashed lines), it extends pseudopods toward the outside of the boundary but cannot adhere to the substrate, owing to which it retracts the pseudopods and then migrates along the boundary.

Next, we quantitatively examined the correlations between the entering and exiting angles of migrating cells when they reached the boundary (Fig 5B). We examined the cell behaviors when cells reached three types of boundaries: linear, concave arc, and convex arc. When entering the linear boundary at a lower angle (θ ≤ 30°), they tended to migrate along the boundary. When reaching the linear boundary at a higher angle (60° < θ ≤ 90°, relatively perpendicular to the boundary), they tended to migrate away at a higher angle (Fig 5C). When reaching the concave or convex arc boundary (radius = 100 μm), they tended to migrate along the boundary at any entering angle (Fig 5D and 5E).

In Fig 5F and 5G, we show typical phase-contrast and fluorescence images of cells expressing GFP-lifeact within the circle (cell number, 200; area density, 32%). Fig 5H presents an averaged image of GFP-lifeact, captured during 1 h of cell migration, suggesting that the fluorescence intensities in the outermost region near the boundary are much higher. Fig 5I presents results of a quantitative analysis of the averaged fluorescence intensity of the areas that were divided into four concentric regions (red, yellow, blue, and green in Fig 5H): the cells exhibited a tendency to be located primarily outermost region near the boundary (green). This is consistent with the above observation that cells tend to migrate along the boundary of the adhesive area. Presumably, the cells migrate along the boundary in search of the adhesive site.

## Discussion

In this study we have described two novel methods for confining cells within an adhesion area of a desired size and shape. Methods similar to those of the first method have been reported; for example, deep UV (<200 nm) irradiation through a photomask onto the surface of an untreated polystyrene plastic dish has been shown to produce patterning of cells [35], but cell movement is not limited to the irradiated area. In Cytograph, a more reliable photoreactive PEG-coated coverslip, exposed to UV light through a photomask, is used [36]. A similar method using PEG gel has been applied for local adhesion experiments for *Dictyostelium* cells; similar to the current study, the cell movement is not limited to the adhesive area [37]. Other non-adhesive coating materials susceptible to UV light, such as polyvinyl alcohol and polyacrylamide, have also been used instead of PEG [38, 39]. These materials are non-adhesive but are not sufficient to completely inhibit adhesion. Lipidure, used in the present study, is an excellent non-adhesive material compared to previously used materials [31]. Our proposed method does not require adhesion proteins, which are not essential for many species of cells; therefore, unwanted effects caused by these proteins can be avoided. Therefore, the present method is superior to other conventional methods for ensuring adherence of a broad array of cell types to defined areas.

The second method stems from our recently invented laserporation technique, which can make a pore in the cell membrane and introduce extracellular substances into the cell [25–27, 40, 41]. In this technique, a laser beam is focused on a small local spot beneath a single cell that is attached to a coverslip, the surface of which was coated with carbon, and the energy of the laser beam absorbed by the carbon generates a small pore in the cell membrane. In the present study, we used gold, instead of carbon, for the coating. Absorption of the laser beam energy generates heat in the carbon coating; in the gold coating, in addition to heat, it generates plasmons. Consequently, this energy removes the Lipidure coat, as evidenced in the results related to the method we have described in this study. Without gold or carbon coating, the same laser power did not remove the Lipidure. When using a gold coating, the 100 mW power of the CW laser was sufficient, in contrast to the very high power of a more expensive pulse laser, which may remove the Lipidure without gold or carbon coating. Although our second method can generate adhesive areas of any shape, it takes several minutes to generate a single area, owing to the limit of laser scanning speed; contrarily, using the first method, we can generate many adhesive areas at a time using a mask that has multiple holes.

Using the two methods described in this study, it was possible to observe and follow individual cells for a long time. We found that the doubling times varied considerably among individual cells, and the division times varied between individual sibling pairs. Previously, these observations were very difficult, especially for fast-moving cells.

The average velocity of the cells did not change when they collided with one another, even at a higher cell density. *Dictyostelium* cells do not seem to show contact inhibition of locomotion, such as that observed in animal cells [42, 43]. Cell–cell adhesion is not fixed, rather it is highly dynamic in *Dictyostelium* cells. When a migrating cell collides with other cells at a lower angle, it frequently changes its direction, reflecting against the other cell. This phenomenon is similar to the collision of keratocytes [44]. However, when *Dictyostelium* cells enter collision at a higher angle, they frequently push or pass through other cells. These behaviors may facilitate the dispersion of cells to cover the surface of the substrate.

Generally, cells migrate on the substrate by alternating four steps: extension (pseudopod) at the anterior, establishment of new adhesions to the substrate at the anterior, forward translocation of the cell body, and detachment of the posterior adhesions by the posterior contraction. After the establishment of new adhesions, cells exert traction force by actin assembly at the anterior and exert posterior contraction via actomyosin contraction, which promotes cell advancement [45–47]. When cells encounter the boundary of the adhesive area, they extend pseudopods toward the outside of the boundary but cannot establish adhesion to the substrate outside the circle; thus, the pseudopods are retracted and the cell eventually migrates along the boundary. Pseudopods or filopods at the anterior of migrating cells may function as sensors for substrate adhesiveness, and thus, determine the direction of cell migration. These structures have been reported to sense the stiffness of the substrate when establishing the adhesion [48, 49] but they might also sense the adhesiveness of the substrate.

In this study, we noted that when the cells neared the linear boundary at a higher angle, they tended to migrate away at a similar angle. However, when cells encountered a linear boundary at a lower angle, they tended to migrate along the boundary. The latter tendency became more pronounced, especially when the cells encountered arced boundaries. This tendency explains the observation that cells are frequently positioned at the outermost region near the boundary of the circular adhesive area.

In conclusion, the methods designed in this study can help researchers track individual single cells for a longer duration, regulate cell density, generate a cell microarray, and generate cell sheets for transplants. When the size of the pattern is set to be very small, it is possible to artificially control cell shape and cytoskeletal organization at the single-cell level.

## Supporting information

S1 Movie Cell adhesion on the fabricated substrates. Suspension of *Dictyostelium* cells was placed on the surface with circular adhesive areas (100 μm in diameter). Cells attached only to adhesive areas, and the unattached cells showed Brownian motion.
(AVI)
